# Giant solitary fibrous tumour of the liver

**DOI:** 10.1186/1477-7819-4-81

**Published:** 2006-11-21

**Authors:** Türkan Terkivatan, Mike Kliffen, Johannes HW de Wilt, Albertus N van Geel, Alexander MM Eggermont, Cornelis Verhoef

**Affiliations:** 1 Department of Surgical Oncology, Erasmus University Medical Center, Daniel den Hoed Cancer Center, Rotterdam, The Netherlands; 2 Department of Pathology, Erasmus University Medical Center, Daniel den Hoed Cancer Center, Rotterdam, The Netherlands

## Abstract

**Background:**

Solitary fibrous tumour (SFT) is an uncommon mesenchymal neoplasm that most frequently affects the pleura, although it has been reported with increasing frequency in various other sites such as in the peritoneum, pericardium and in non-serosal sites such as lung parenchyma, upper respiratory tract, orbit, thyroid, parotid gland, or thymus. Liver parenchyma is rarely affected. Clinically, SFTs cause symptoms after having reached a certain size or when vital structures are involved. In recent years, SFTs are more often identified and distinguished from other tumours with a similar appearance due to the availability of characteristic immunohistochemical markers.

**Case presentation:**

In this manuscript we report the case of a large tumour of the liver, which was histologically diagnosed as a SFT, and showed involvement of a single hepatic segment. Because of the patient's presentation and clinical course, it may represent a radiation-induced lesion.

**Conclusion:**

When a SFT has been diagnosed, surgery is the treatment of choice. The small number of patients with a SFT of the liver and its unknown natural behaviour creates the need to a careful registration and follow-up of all identified cases

## Background

Solitary fibrous tumour (SFT) is an uncommon mesenchymal neoplasm that most frequently affects the pleura [1,2], although it has been reported with increasing frequency in various other sites such as in the peritoneum [3], in the pericardium [4] and in non-serosal sites such as lung parenchyma [5], upper respiratory tract [6], orbit [7], thyroid [8], parotid gland [9], or thymus [10]. Liver parenchyma is rarely affected [11]. Clinically, SFTs cause symptoms after having reached a certain size or when vital structures are involved. In recent years, SFTs are more often identified and distinguished from other tumours with a similar appearance due to the availability of immunohistochemical markers [12,13].

In this manuscript we report the case of a large tumour of the liver, which was histologically diagnosed as a SFT, and showed involvement of a single hepatic segment. Because of the patient's presentation and clinical course, it may represent a radiation-induced lesion.

## Case presentation

**Figure 1:**
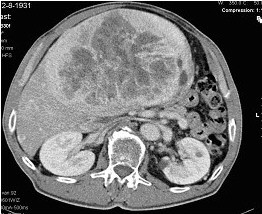
CT-scan of the liver preoperative.

A 74-year-old man was referred to our department with complains of gastric fullness, frequent episodes of post-prandial nausea, and weight loss over the previous 3 months. Medical history revealed a high-grade mucosa-associated lymphoid tissue (MALT) lymphoma of the stomach in 1996. The lymphoma was staged as IE according to the Ann Arbor staging system, and showed complete response after chemotherapy followed by radiation therapy (60 Gy) of the local nodal regions. On physical examination, a large mass was palpated in the epigastrium that filled the entire right upper quadrant of the abdomen. Routine laboratory investigations, including blood glucose levels, and serum levels of tumour markers (alpha-fetoprotein [AFP], carcinoembryonic antigen [CEA], and carbohydrate antigen [Ca19-9]) were within normal limits. Computed tomography (CT) of the abdomen demonstrated a large, heterogeneous mass in the left hepatic lobe, measuring 24 cm in largest diameter, with necrosis and calcification (Figure 1). There were no signs of recurrence of high grade MALT lymphoma. Liver biopsy showed spindle-cell elements with cellular atypia suggesting a sarcoma. Laparotomy was performed and revealed a large tumour, which only involved segment IV of the liver, and a left lobectomy was performed. Postoperative course was uneventful. After 12 months of follow-up the patient had no signs of local recurrence or distant metastases.

### Pathologic examination

**Figure 2:**
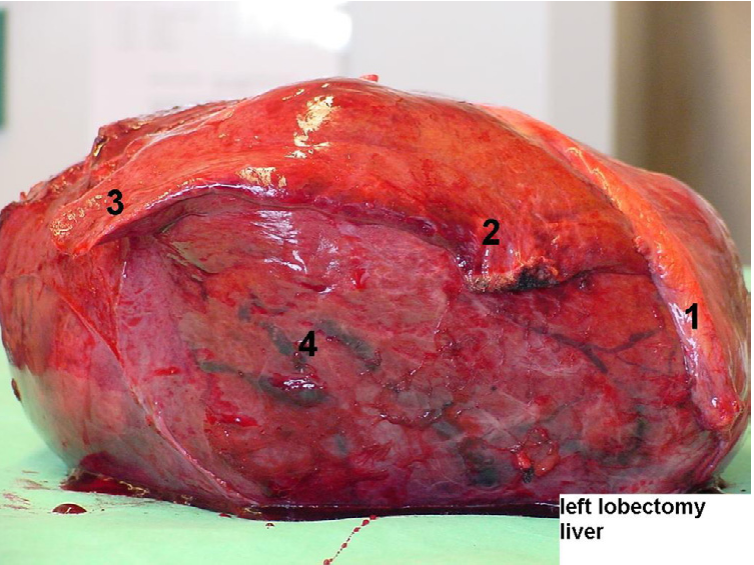
Left Lobectomy specimen. Large tumour situated in segment IV, atrophy of segments II and III. 1: falciform ligament, 2: segment II, 3: segment III, 4: segment IV (tumour).

**Figure 3:**
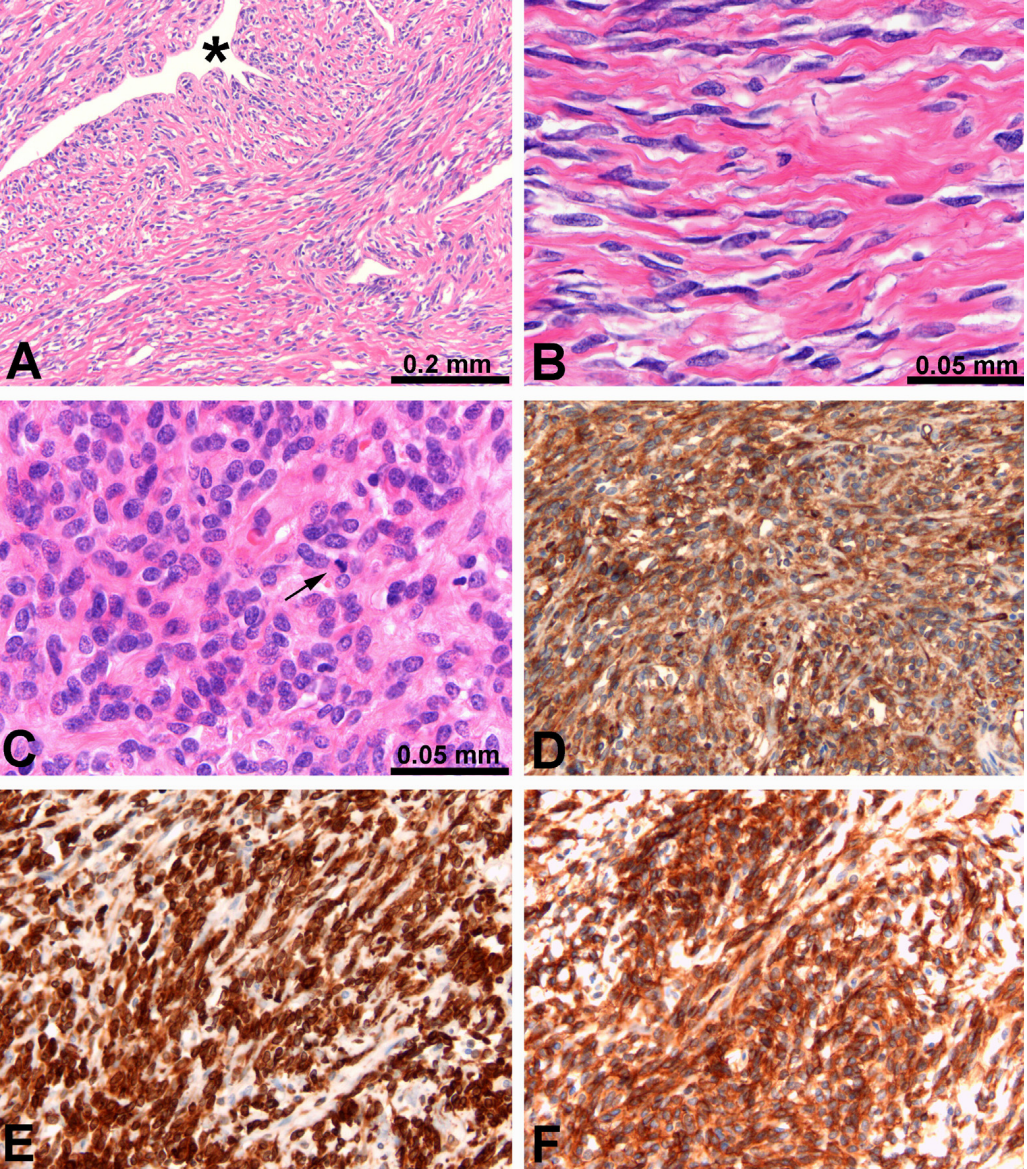
Light microscopy. **A **Characteristic picture of the tumour showing spindle cells with surrounding hyalinized collagen bundles, and haemangiopericytoma-like vascular space (*). **B **Higher magnification of spindle cell area with intermixed collagen. **C **Higher magnification of cellular area with little collagen and increased amount of mitoses (arrow). **D, E, and F **Immunohistochemical staining for CD34, BCL-2, and CD99, respectively.

Gross examination showed a large tumour measuring 24 × 21 × 15 cm that weighed 2950 g (Figure 2). Macroscopically, the tumour was located in the liver parenchyma and not in relation to serosal surface. The lesion was well encapsulated, and contained a number of central necrotic and scarred areas with radiating bands of fibrous tissue. Microscopically, the tumour was composed of neoplastic spindle cells, with uniform, elongated nuclei, separated by abundant thick bands of collagen (Figure 3a, and 3b). A few highly cellular areas with high mitotic rate of 10 – 13 mitosis/10 HPF were detected. In the higher cellular areas, cells were close together with limited amounts of collagen (Figure 3c). The organization of the tumour cells was different in several areas, varying from small bundles of spindle cells arranged haphazardly, arranged in some groups with nuclear pallisading or in a storiform pattern. Cells were separated by an abundant reticulin network, which appeared in a random pattern. An abundant thin walled vascular network was present with some heamangiopericyoma-like vascular spaces (Figure 3a). Immunohistochemistry revealed strong expression of CD34, CD99, BCL2 and vimentin in virtually all tumour cells (Figure 3d,3e, and 3f). No immunoreactivity was shown with pankeratin, keratin 8/18, epithelial membrane antigen, smooth-muscle actin, desmin, S100, Factor VIII, CD 31 and CD117. Epidermal growth factor receptor (EGFR) (100%) and progesterone receptor (10%–20%) were positive. Oestrogen receptor (ER) and Her-2 neu were negative.

## Discussion

The possible relationship between irradiation and neoplasia is well known. Radiation-induced soft tissue tumours are uncommon and mostly high-grade lesions, such as angiosarcoma after irradiation of the breast [14,15]. SFT of the liver has not been described in the context of radiation, but it was reported that radiation of the liver may cause an inflammatory reaction and fibrosis which may cause radiation-induced liver disease [16]. In the presented case; the tumour was developed in the documented field of irradiation, the tumour was detected 9 years after exposure and chronic lymphedema was not present in the liver. Our patient satisfies all the three criteria to consider a tumour to be irradiation related [17]. Since the mid-to-late 1990s positive immunohistochemical staining for CD34 and vimentin, along with a haemangiopericytoma-like appearance, became the hallmark of SFTs [12,13]. Nowadays, the monoclonal antibody CD34 has received a wide consensus as a diagnostic marker for SFT, and an increasing number are reported in the literature [11]. Although immunoreactivity with CD34 is believed to be highly characteristic of SFTs, and a positive CD34 staining is required for diagnosis, the immunohistologic staining pattern is not entirely specific [18]. Other entities, such as angiosarcoma and gastrointestinal stromal tumour, also express CD34 and vimentin epitopes [19,20]. Therefore, other histomorphological and immunohistochemical characteristics remain evenly important. In the present case, CD117 negativity, CD31 negativity, Factor VIII negativity combined with the histomorphology, excluded gastrointestinal stromal tumour and angiosarcoma. The strong expression of CD99 and BCL2 in the tumour cells further supports the diagnosis of SFT, but as stand-alones these markers would not be sufficient.

Histologically, the alternating hypercellular and hypocellular areas and a hemangiopericytoma-like vascular pattern with abundant branching of thin-walled vessels dissecting through the tumour, are typical findings in benign and malignant SFT of the liver [21,22]. The focal presence of intense hypercellularity accompanied by increased nuclear atypia and an elevated mitotic rate are criteria for malignant change, as is necrosis. There is also some evidence that the rate of mitosis as well as the size of the tumour might well be possible predictors of local recurrence and metastatic disease [21,23]. These malignant elements may not be detected on a needle core biopsy due to the hazard of sampling error, and a histopathological examination of the resected specimen is needed to predict clinical course.

When a SFT has been diagnosed, surgery is the treatment of choice. Although not a primarily malignant disease, it seems to be of importance to achieve tumour-free margins in order to prevent local recurrence or distant metastasis [21,23]. Due to the infrequent prevalence of SFT, malignant potential and recurrence rate can not be determined exactly. According to the literature, intra-thoracic SFTs show a local recurrence and distant metastatic rate of up to 15%, while extra-thoracic tumours may demonstrate malignant behaviour in approximately 6% [11].

## Conclusion

When a SFT has been diagnosed, surgery is the treatment of choice. The small number of patients with a SFT of the liver and its unknown natural behaviour creates the need to a careful registration and follow-up of all identified cases. Because of the patient's presentation and clinical course in our case, it may represent a radiation-induced lesion.

## Competing interests

The author(s) declare that they have no competing interests.

## Authors' contributions

**CV **and **JHW **carried out the surgical procedure and contributed to the design of the study. **TT **and **CV **gathered the data from the literature search and made the first draft of the manuscript. **MK **performed the histological analysis of the surgical specimen and provided histological sections as figures for the manuscript. **ANG**, **AMME **and **JHW **revised and finally approved the manuscript for been published. All authors approved the final manuscript.
